# Genome Sequences of Two NDM-1 Metallo-β-Lactamase-Producing Multidrug-Resistant Strains of *Klebsiella pneumoniae* with a High Degree of Similarity, One of Which Contains Prophage

**DOI:** 10.1128/genomeA.01173-17

**Published:** 2017-10-19

**Authors:** Nikolay V. Volozhantsev, Angelina A. Kislichkina, Anastasia I. Lev, Tatiana N. Mukhina, Alexander A. Bogun, Olga N. Ershova, Irina A. Alexandrova, Nadezhda K. Fursova

**Affiliations:** a State Research Center for Applied Microbiology and Biotechnology, Obolensk, Moscow Region, Russia; b Burdenko Neurosurgical Institute, Moscow, Russia

## Abstract

We report genome sequences of two NDM-1 metallo-β-lactamase-producing multidrug-resistant *Klebsiella pneumoniae* isolates of sequence type 147 (ST147) from one hospital. The genomes are highly similar and differ in prophage located in the chromosome of *K. pneumoniae* KPB-1470/16 and in the additional plasmid-carrying *bla*_OXA-48_ gene in *K. pneumoniae* KPB-417/16.

## GENOME ANNOUNCEMENT

*Klebsiella pneumoniae* carrying New Delhi metallo-β-lactamase (NDM-1) emerging worldwide has raised public health concern. NDM-1 hydrolyzes a wide range of β-lactam antibiotics, including carbapenems, which are the last-resort antibiotics for the treatment of infections caused by resistant bacteria. Nosocomial infections caused by carbapenem-resistant *K. pneumoniae* are associated with high rates of morbidity and mortality (1, 2). This pathogen was included in the critical level of the “global priority list of antibiotic-resistant bacteria” designed by the World Health Organization (3). The dissemination of NDM-1 is associated with diverse sequence types (STs) of *K. pneumoniae*, including ST147 (2, 4, 5).

Here, we report the genome sequences of two ST147 *K. pneumoniae* strains (KPB-1470/16 and KPB-417/16) isolated from endotracheal aspirates of two adult patients in the Moscow neurosurgical intensive care unit (ICU) on 28 March 2016 and 5 September 2016, respectively. The strains were deposited in the State Collection of Pathogenic Microorganisms and Cell Cultures “SCPM-Obolensk” (accession numbers SCPM-O-B-8045 and SCPM-O-B-7954, respectively). Both strains are resistant to amoxicillin-clavulanic acid, ampicillin-sulbactam, cefuroxime, ceftazidime, cefoperazone-sulbactam, cefepime, imipenem, ciprofloxacin, gentamicin, amikacin, and nitrofurantoin.

Genome sequencing was performed using an Illumina MiSeq instrument according to the manufacturer’s instructions. For each genome, reads were assembled *de novo* using SPAdes v. 3.9.0 (6). The final assemblies had mean coverages of 36× and 43× and consisted of 5,625,359 bp (GC content of 57.0% and 115 contigs) and 5,637,851 bp (GC content of 57.0% and 110 contigs) for KPB-417/16 and KPB-1470/16, respectively.

Draft genomes were annotated using the NCBI Prokaryotic Genome Annotation Pipeline (7). A total of 5,367 and 5,399 protein-coding sequences and 106 and 109 tRNAs were annotated for KPB-417/16 and KPB-1470/16, respectively. Sequences of four plasmid replicon types (IncHIIB, IncFIA, IncFIB, and IncFII) were identified in both genomes by using PlasmidFinder (8). In addition, sequences of IncL/M plasmid were determined in the KPB-417/16 genome. Six different *bla* genes (*bla*_SHV-11_, *bla*_CTX-M-15_, *bla*_TEM-1_, *bla*_OXA-1_, *bla*_OXA-9_, and *bla*_NDM-1_) that code for extended-spectrum β-lactamase were defined in both genomes using ResFinder (9). An additional *bla*_OXA-48_ gene was identified in IncL/M plasmid sequences of *K. pneumoniae* KPB-417/16. Moreover, genes that determine resistance to aminoglycosides [*aadA1*, *aadA2*, *armA*, *aph*(*3′*)*-via*, and *aac*(*6′*)*Ib-cr*], fluoroquinolones (*oqxAB*), phosphomycins (*fosA*), macrolides [*msr*(*E*) and *mph*(*E*)], phenicols (*catB4*), sulfonamides (*sul1*), and trimethoprim (*dfrA12*) have been identified in chromosome and plasmid sequences of both genomes. Some resistance genes [*aac*(*3*)*-IIa*,* qnrS1*,* and catA2*] were determined in plasmid sequences of strain KPB-1470/16 only.

Both strains exhibit a high average nucleotide identity of 99.99% between each other but differ from one another in the plasmid composition mentioned above as well as by the presence of additional prophage sequences located in the chromosome of *K. pneumoniae* KPB-1470/16. Similar prophage sequences were detected in the genome of *K. pneumoniae* strain TGH13 (GenBank accession number CP012745) isolated in Greece that belongs to ST147 as well.

According to our knowledge, this is the first report of genome sequences of NDM-1 metallo-β-lactamase-producing strains of *K. pneumoniae* isolated in Russia. The characteristics of the presented genomes are a step toward a better understanding of the population of clinical multidrug-resistant *K. pneumoniae* strains.

### Accession number(s).

This whole-genome shotgun project has been deposited at DDBJ/EMBL/GenBank under the accession numbers NPJW00000000 and NPII00000000 for strains KPB-417/16 and KPB-1470/16, respectively.

## References

[1] Arnold RS, Thom KA, Sharma S, Phillips M, Johnson JK, Morgan DJ 2011 Emergence of *Klebsiella pneumoniae* carbapenemase (KPC)-producing bacteria. South Med J 104:40–45. doi:10.1097/SMJ.0b013e3181fd7d5a. 21119555 PMC3075864

[2] Pitout JD, Nordmann P, Poirel L 2015 Carbapenemase-producing *Klebsiella pneumoniae*, a key pathogen set for global nosocomial dominance. Antimicrob Agents Chemother 59:5873–5884. doi:10.1128/AAC.01019-15. 26169401 PMC4576115

[3] World Health Organization. 2017 Global priority list of antibiotic-resistant bacteria to guide research, discovery, and development of new antibiotics. http://www.who.int/entity/medicines/publications/WHO-PPL-Short_Summary_25Feb-ET_NM_WHO.pdf?ua=1.

[4] Shin J, Baek JY, Cho SY, Huh HJ, Lee NY, Song JH, Chung DR, Ko KS 2016 bla_NDM-5_-bearing IncFII-type plasmids of *Klebsiella pneumoniae* sequence type 147 transmitted by cross-border transfer of a patient. Antimicrob Agents Chemother 60:1932–1934. doi:10.1128/AAC.02722-15. 26824953 PMC4775946

[5] Messaoudi A, Haenni M, Mansour W, Saras E, Bel Haj Khalifa A, Chaouch C, Naija W, Boujâafar N, Bouallègue O, Madec JY 2017 ST147 NDM-1-producing *Klebsiella pneumoniae* spread in two Tunisian hospitals. J Antimicrob Chemother 72:315–316. doi:10.1093/jac/dkw401. 27659734

[6] Bankevich A, Nurk S, Antipov D, Gurevich AA, Dvorkin M, Kulikov AS, Lesin VM, Nikolenko SI, Pham S, Prjibelski AD, Pyshkin AV, Sirotkin AV, Vyahhi N, Tesler G, Alekseyev MA, Pevzner PA 2012 SPAdes: a new genome assembly algorithm and its applications to single-cell sequencing. J Comput Biol 19:455–477. doi:10.1089/cmb.2012.0021. 22506599 PMC3342519

[7] Tatusova T, DiCuccio M, Badretdin A, Chetvernin V, Nawrocki EP, Zaslavsky L, Lomsadze A, Pruitt KD, Borodovsky M, Ostell J 2016 NCBI Prokaryotic Genome Annotation Pipeline. Nucleic Acids Res 44:6614–6624. doi:10.1093/nar/gkw569. 27342282 PMC5001611

[8] Carattoli A, Zankari E, García-Fernández A, Voldby Larsen M, Lund O, Villa L, Møller Aarestrup F, Hasman H 2014 *In silico* detection and typing of plasmids using PlasmidFinder and plasmid multilocus sequence typing. Antimicrob Agents Chemother 58:3895–3903. doi:10.1128/AAC.02412-14. 24777092 PMC4068535

[9] Zankari E, Hasman H, Cosentino S, Vestergaard M, Rasmussen S, Lund O, Aarestrup FM, Larsen MV 2012 Identification of acquired antimicrobial resistance genes. J Antimicrob Chemother 67:2640–2644. doi:10.1093/jac/dks261. 22782487 PMC3468078

